# A case report of thin basement membrane nephropathy accompanied by sporadic glomerulocystic kidney disease

**DOI:** 10.1186/s12882-019-1451-6

**Published:** 2019-07-09

**Authors:** Hiroyuki Hashimoto, Naro Ohashi, Naoko Tsuji, Yoshitaka Naito, Shinsuke Isobe, Tomoyuki Fujikura, Takayuki Tsuji, Akihiko Kato, Kandai Nozu, Kazumoto Iijima, Hideo Yasuda

**Affiliations:** 1 Internal Medicine 1, Hamamatsu University School of Medicine, 1-20-1 Handayama, Higashi-ku Hamamatsu, 431-3192 Japan; 2 Blood Purification Unit, Hamamatsu University School of Medicine, 1-20-1 Handayama, Higashi-ku Hamamatsu, 431-3192 Japan; 3 Department of Pediatrics, Kobe University Graduate School of Medicine, 7-5-1 Kusunoki-cho, Chuo-ku, Kobe, 650-0017 Japan

**Keywords:** Thin basement membrane nephropathy, Glomerulocystic kidney disease, Renal biopsy, Genetic testing, Case report

## Abstract

**Background:**

Thin basement membrane nephropathy (TBMN) is a relatively common disease. Patients typically present with isolated hematuria, which has a good renal prognosis. In contrast, glomerulocystic kidney disease (GCKD) is a rare disease, associated with slow progressive renal dysfunction. To our knowledge, co-occurring diagnosis of TBMN with GCKD has not been reported previously.

**Case presentation:**

A 30-year old woman was admitted to our hospital for evaluation of hematuria and renal insufficiency. Upon examination, her urinary protein level was 40 mg/day and occult blood in her urine was 2+. The patient’s urinary dysmorphic red blood cell sediment was 30–49/high power field. In contrast, her serum creatinine levels increased from 0.57 mg/dl to 0.86 mg/dl during the previous 2-years, without special events. She suffered from far-sightedness and astigmatism beginning at birth; She had no family history of renal disease. Renal biopsy demonstrated cystic dilatation of the Bowman’s capsule and atrophy of the glomerular tuft. The glomerular basement membrane (GBM) was thin, with an average thickness of 191 nm. Next-generation sequencing was used to evaluate for mutations in *COL4A3* and *COL4A4*, associated with TBMN, and *UMOD, MUC1*, and *SEC61A1*, associated with hereditary GCKD. No pathogenic mutations were identified. We thus diagnosed the patient with TBMN coexistent with sporadic GCKD.

**Conclusion:**

We report the patient diagnosed with TBMN accompanied by sporadic GCKD, based on renal biopsy and genetic testing. Because it is possible that other diseases, such as GCKD, can coexist with TBMN, it is important to consider renal biopsy.

## Background

Thin basement membrane nephropathy (TBMN) is a disease characterized by thinning of the glomerular basement membrane (GBM) mainly caused by gene mutations of type IV collagen α3 or α4 (*COL4A3* or *COL4A4*), which are the component materials of the GBM [1]. Haas et al. established criteria for the lower limit of normal GBM thickness as 230 nm in male patients and 215 nm in female patients at the age of 9-years or older [1].

TBNM is one of the most frequent causes of isolated microscopic hematuria, with an incidence of approximately 1% in the general population [2, 3]. In general, urinary protein excretion and blood pressure are normal in patients with TBMN while the characteristic manifestation is persistent or intermittent asymptomatic microscopic hematuria [4].

It is well established that TBMN coexists with other glomerular pathologies. Qazi et al. reported a TBMN prevalence of 47 (7.4%) in a total of 634 biopsies. Among these cases, 17 (36.2%) had TBMN alone and the remaining 30 (63.8%) were patients with other glomerular pathologies, as follows: immunoglobulin A (IgA) nephropathy, 9 (19.1%); focal segmental glomerulosclerosis (FSGS), 9 (19.1%); pauciimmune crescentic glomerulonephritis, 3 (6.4%); mesangioproliferative glomerulonephritis, 2 (4.3%); acute interstitial nephritis, 2 (4.2%); lupus nephritis, 1 (2.1%); focal endocapillary proliferative glomerulonephritis, 1 (2.1%); acute endocapillary glomerulonephritis, 1 (2.1%), chronic sclerosing glomerulonephritis, 1 (2.1%), and; both IgA nephropathy and FSGS, 1 (2.1%) [5].

In contrast, glomerulocystic kidney disease (GCKD), first reported by Ribbert et al. in 1889, is a rare disease in which cystic dilatation of the Bowman’s capsule and atrophy of the glomerular tuft are observed. Glomerular cysts are defined as Bowman space dilatation greater than 2 to 3 times the normal size. They occur in disorders of diverse etiology and are associated with a spectrum of clinical manifestations. GCKD is diagnosed in patients with greater than 5% cystic glomeruli in the kidneys [6].

Lennerz et al. retrospectively assessed 20 cases of GCKD from their records and identified > 230 cases published in the literature [7]. However, to the best of our knowledge, we believe that this is the first case of TBMN co-occurring with GCKD reported in the literature. Herein, we report on this rare case of a patient with TBMN and sporadic GCKD diagnosed by renal biopsy and genetic testing.

## Case presentation

A 30-year old woman was admitted to our hospital to undergo renal biopsy. According to her medical records, microscopic hematuria was detected on every urine analysis since her birth. At 16-years-old, the potential causes were closely examined. However, no diagnosis was made. Renal biopsy was not performed at that time because the patient’s manifestation was only microscopic hematuria without increased urinary protein or other signs of renal dysfunction. A few years later, she chose to stop her regular visits to the doctor. When she was 27-years old, she became pregnant. Upon her initial visit at the department of obstetrics and gynecology, she tested positive for hematuria. In the 30th week of pregnancy, her urine tested positive for the presence of protein but the results returned to normal after puerperium. Further, the patient experienced hypertension soon after delivery, but her blood pressure was normotensive during all other periods. Through entire pregnancy and post-partum, evidence of hematuria remained upon testing her urine samples.

The patient did not experience any subjective or objective symptoms associated with kidney disorders such as, fever, deafness, hemoptysis, or rash. In addition, she was not taking any medication. Her urinary findings did not change over 2-years. The urinary protein level was 60 and 40 mg/day in 2016 and 2018, respectively. Hematuria was assessed using a urinary occult blood test resulting in 3+ and 2+ in 2016 and 2018, respectively. In addition, the urinary dysmorphic red blood cell (RBC) sediment was 50–99/high power field (HPF) and 30–49/HPF in 2016 and 2018, respectively. In contrast, serum creatinine levels increased from 0.57 mg/dl to 0.86 mg/dl during the same 2-years, without special events.

The patient had a history of far-sightedness, astigmatism, and strabismus from birth as well as polycystic ovarian syndrome diagnosed at the age of 27-years. Her family history included a paternal grandmother with a subarachnoid hemorrhage and a father with hypertension. There was no family history of renal dysfunction or urinary abnormalities.

Physical examination results on admission were as follows: height 152 cm, weight 42.0 kg, body mass index (BMI) 18.2 kg/m^2^, body temperature 36.8 °C, blood pressure 99/76 mmHg, and pulse of 73 beats/min. Hearing loss, skin manifestations, and joint symptoms were not detected. In addition, the patient’s respiratory sounds were normal and abdominal tenderness was not detected. Laboratory data showed renal insufficiency [serum creatinine, 0.86 mg/dl; estimated glomerular filtration rate (eGFR), 64 ml/min/1.73 m^2^] and hematuria (urinary occult blood, 3+; urinary dysmorphic RBC sediment, 11.3/HPF) (Table 1). eGFR was calculated by serum creatinine concentrations using the Japanese eGFR equation [8]. The patient’s urinary protein level was 60 mg/day, which is classified as normal to mildly increased proteinuria according to the Kidney Disease: Improving Global Outcomes 2012 Clinical Practice Guidelines [9]. Immunological testing showed no abnormal findings (Table 1).

**Table 1:** Laboratory findings on admission

Parameter	Value (reference range)
Hematology	
WBC count, /μL	5630 (3000–9000)
Hemoglobin, g/dL	10.5 (11.0–15.0)
Platelet count, 10^4^/μL	21.9 (14.5–35.0)
Blood chemistry	
SCr, mg/dL	0.86 (0.2–0.8)
eGFR, mL/min/1.73 m^2^	64 (> 90)
SUN, mg/dL	13.3 (8–22)
Serum albumin, g/dL	4.3 (3.9–4.9)
AST, U/L	16 (13–30)
ALT, U/L	7 (10–40)
LDH, U/L	162 (110–210)
HbA1c, %	5.2% (4.6–6.2)
FBS, mg/dl	93 (70–109)
Triglyceride, mg/dl	80 (50–149)
T. cholesterol, mg/dl	166 (150–219)
HDL-C, mg/dl	40 (40–06)
FDP-DD, μg/ml	< 1.0 (< 1.0)
PT-INR	1.00 (0.9–1.10)
Immunology	
CH50, U/ml	44 (31–49)
C3, mg/dL	77 (75–148)
C4, mg/dL	21 (14–38)
ANA	< 40 (< 40)
Anti-dsDNA antibody, IU/mL	< 10 (< 12)
MPO-ANCA, U/mL	< 1.0 (< 3.5)
PR3-ANCA, U/mL	< 1.0 (< 3.5)
Rheumatoid factor, IU/mL	5.2 (0–15)
HIV antibody	Negative
HCV antibody	Negative
HBV surface antigen	Negative
HBV surface antibody	Negative
Urinalysis	
Urine occult blood	2+
Urine dipstick protein	–
Urine RBC/HPF	11.3 (0.0–4.9)
Urine WBC/HPF	0.7 (0.0–5.0)
Spot urine PCR, g/g	0.04 (< 0.15)

Abbreviations: *ALT,* Alanine aminotransferase, *ANA,* Antinuclear antibody; *ANCA*, Antineutrophil cytoplasmic antibody, *AST*, Aspartate aminotransferase, *CH50*, Complement activity, *dsDNA*, Double-stranded DNA, *eGFR*, Estimated glomerular filtration rate, *FBS*, Fasting blood sugar, *FDP-DD*, Fibrin degradation products-*D*, Dimer, *HbA1c*, Hemoglobin A1c, *HBV*, Hepatitis B virus, *HCV*, Hepatitis C virus, *HDL-C,* High density lipoprotein cholesterol, *HIV*, Human immunodeficiency virus, *HPF*, High power field, *INR,* International normalized ratio, *LDH*, Lactate dehydrogenase, *MPO*, Myeloperoxidase, *PCR,* Protein-creatinine ratio, *PR3*, Proteinase 3, *PT*, Prothrombin time, *RBC,* Red blood cell, *SCr*, Serum creatinine, *SUN*, Serum urea nitrogen, *T*, Cholesterol total cholesterol, *WBC*, White blood cell

**Fig. 1:**
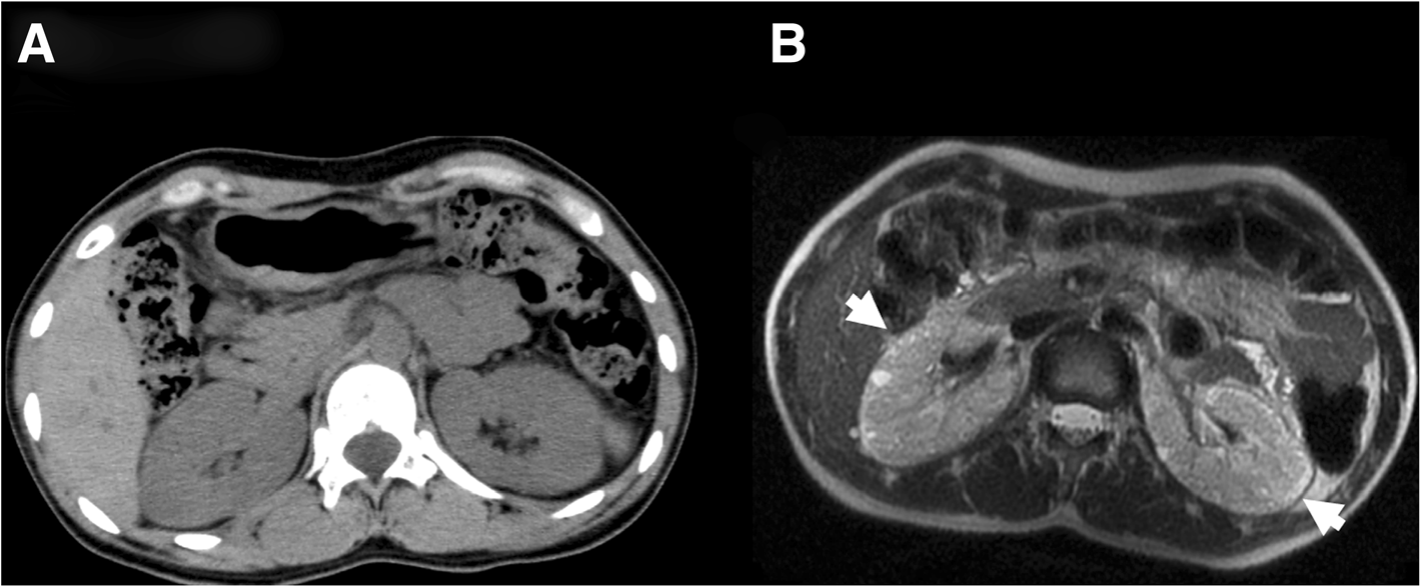
Kidney morphology. **a** Computed tomography scan of the kidney. The density of the renal cortex was remarkably low in the bilateral, normal sized kidneys. **b** Magnetic resonance imaging (MRI) of the kidneys. Numerous small high intensity spots (arrow) were distributed within the subcapsular cortex on T2-weighted MRI

The kidney size was within normal limits (right: 109 × 58 mm; left, 108 × 62 mm) and compression of the left renal vein between the aorta and proximal superior mesenteric artery, which would suggest Nutcracker syndrome, was not detected on abdominal computed tomography (CT). However, the density of the renal cortex was remarkably low (Fig. 1 a). In addition, numerous small high-intensity spots were distributed within the subcapsular cortex on magnetic resonance imaging T2-weighted image (Fig. 1 b).

**Fig. 2:**
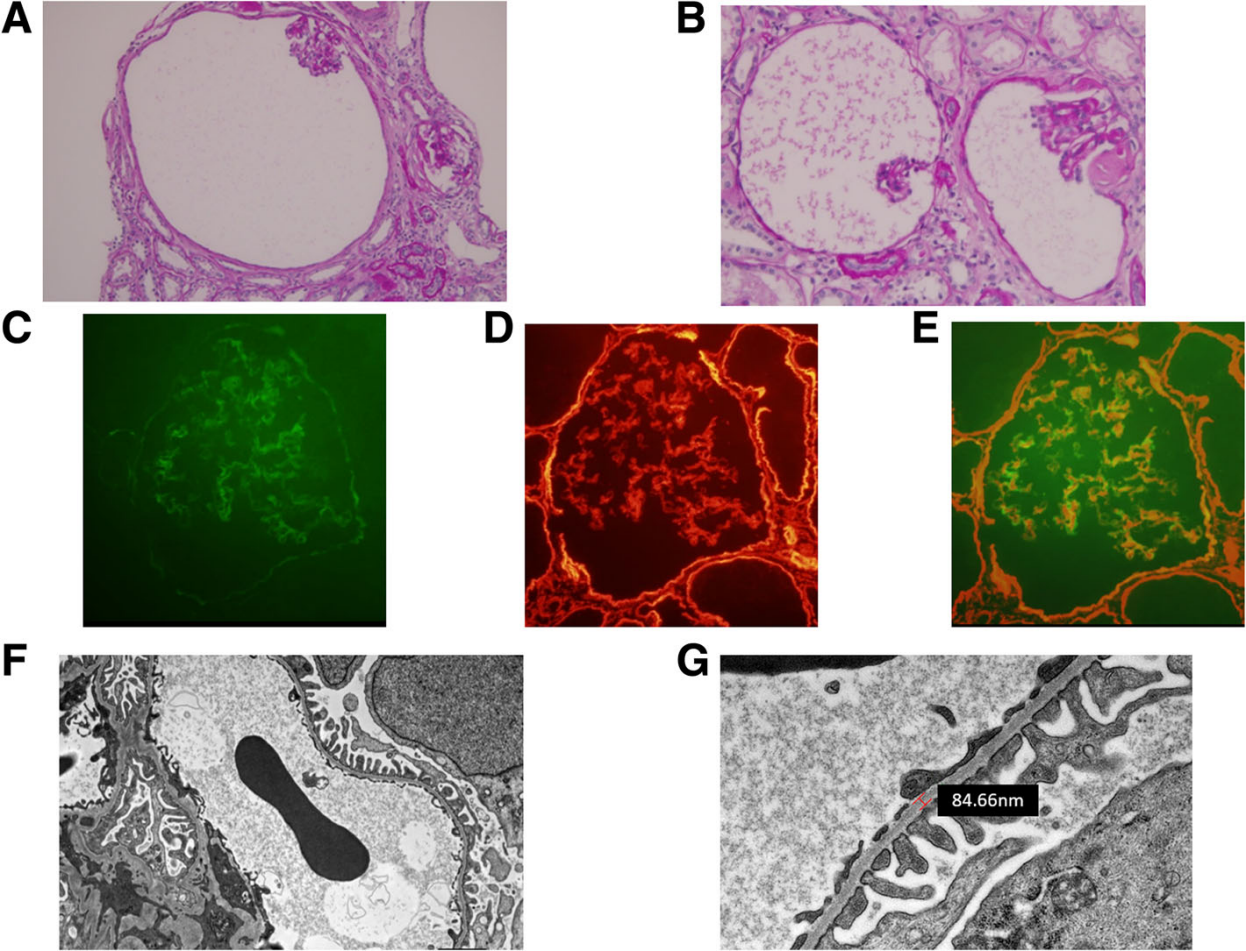
Renal biopsy findings. *Light microscopy.* The glomerulus shows cystic dilatation of Bowman’s capsule and atrophy of the glomerular tuft, (1A) (periodic acid-Schiff (PAS) stain: original magnification × 100) and (1B) (PAS stain: original magnification × 400). *Type IV collagen staining.* Immunofluorescence study was performed to evaluate the expression levels of type IV collagen. FITC-conjugated-anti α5 chain is observed in the glomerular basement membrane, part of the tubular basement membrane, and Bowman’s capsular basement membrane (1C). In addition, Texas Red-anti α2 chain revealed the renal basement membrane structure (1D). Basement membranes where two fluorescence were present appear green to yellow (1E) (original magnification × 400). Fluorochrome-conjugated anti collagen IV cocktail for Alport’s syndrome was used (Cosmo Bio Ltd. Co., Tokyo, Japan). *Electron microscopy.* Morphological abnormalities, apart from thinning of the glomerular basement membrane, were not found; there were no electron dense deposits (1F; original magnification × 3000 and 1G; original magnification × 10,000)

The differential diagnosis of hematuria from early childhood include IgA nephropathy, Alport syndrome, and TBMN. Therefore, we performed renal biopsy the day after admission. Renal histological examination revealed global sclerosis in 5 of 22 glomeruli, cystic dilatation of the Bowman’s capsule in 3 of 22 glomeruli (14.1%), and atrophy of the glomerular tufts (Fig. 2 a and b). Immunofluorescence studies indicated no deposition of immunoglobulins or complement proteins. Type IV collagen staining showed normal expression levels of the α5 chain in the GBM and Bowman’s capsule (Fig. 2 c to e). The renal biopsy samples obtained for electron microscopy examination did not contain cystic glomeruli and therefore, the detailed appearance of cystic glomeruli could not be evaluated. Electron microscopy revealed thinning of the GBM. The width of the GBM was measured at 30 random points to calculate the average value. The average thickness of the GBM was 191 nm, which exceeded criteria set forth by Haas et al. for the lower limit of normal thickness (215 nm for females) [1] (Fig. 2 f and g). Other morphological abnormalities, including electron-dense deposits and foot process effacement, were not identified on electron microscopy. Based on the above findings, the patient was diagnosed with TBMN accompanied by GCKD.

Although this patient had no recorded family history of GCKD, we examined whether there were any associations with known genetic mutations using next-generation sequencing. There were no pathogenic mutations of *UMOD, MUC1,* or *SEC61A1*, encoding uromodulin, mucin1, or Sec61 translocon alpha 1 subunit, which are causative proteins of autosomal dominant tubulointerstitial kidney disease, including GCKD. No pathogenic mutations of *COL4A3* and *COL4A4*, encoding type IV collagen α3 and α4 of the GBM, were detected. Based on these results, we diagnosed the patient with TBMN coexistent with sporadic GCKD. The patient was discharged and followed closely without intervention.

## Discussion and conclusions

We diagnosed a patient with TBMN accompanied by sporadic GCKD based on negative genetic tests for commonly associated mutations. The patient presented with isolated hematuria without proteinuria since birth. Urinary protein excretion and blood pressure are typically normal in patients with TBMN and the characteristic manifestation of TBMN is persistent or intermittent asymptomatic microscopic hematuria incidentally discovered on routine urinalysis [4]. Therefore, we suspected the diagnosis of TBMN on admission to the hospital. However, the progressive renal dysfunction in the patient was atypical.

Alport syndrome is a genetically heterogeneous condition characterized by structural abnormalities in the GBM. The clinical course of Alport syndrome is typically manifested as hematuria and progressive renal insufficiency developing from childhood to early- or middle-aged adulthood [1]. While our findings reflected those of Alport syndrome, the absence of diseased proteinuria, anterior lenticonus, and hearing loss did not correspond with that diagnosis. IgA nephropathy is the most common glomerulonephritis globally, with a clinical presentation of asymptomatic hematuria. Isolated microscopic hematuria with minimal proteinuria is regarded as a favorable prognosis [10]. However, one factor associated with poor prognosis is proteinuria [11]. In this case, the levels of proteinuria remained normal to mildly increased, despite aggravated renal function. Therefore, it was difficult to confirm IgA nephropathy as a significant differential diagnosis. Hence, we performed a renal biopsy. As a result, we recorded no longitudinal splitting of the lamina densa of the GBM and Alport syndrome was ruled out. In addition, mesangial cell proliferation and mesangial matrix accumulation were not visible, and immunofluorescence studies indicated no deposition of IgA. Therefore, IgA nephropathy was also ruled out.

Angiotensin converting enzyme inhibitors or angiotensin receptor blockers are recommended to prolong renal survival for patients of Alport syndrome or TBMN with or without proteinuria. Although she had no known genetic mutation associated with Alport syndrome or TBMN, angiotensin converting enzyme inhibitors or angiotensin receptor blockers might be her potential option [12–14].

The clinical manifestations of GCKD are divided into early-onset (in neonates) and late-onset (in adults). Renal function in early-onset GCKD follows a stable course for several years and may progress to end-stage renal disease in ≥3-years. Late-onset GCKD typically shows less severe renal impairment [7]. It is possible that superimposed glomerulonephritis [15] or distinct diseases [16] can explain the different clinical manifestations of GCKD.

We were able to diagnose TBMN coexistent with GCKD after reviewing the renal biopsy specimen in this case. This illustrates the importance of renal biopsy when clinical findings do not correlate well with the expected clinical course of renal disease. Since it is possible to treat IgA nephropathy following diagnosis via renal biopsy, the biopsy in this case was meaningful, allowing the patient to be discharged and followed closely without intervention. Therefore, we believe renal biopsy was necessary to define the appropriate treatment or close follow-up.

Because GCKD is a rare disease and 80% cases of GCKD occur in early life, nephrologists seldom encounter adult patients with GCKD, making it difficult to come up with the diagnosis of GCKD. GCKD was divided into 5 categories by Lennerz et al., according to the etiology and spectrum of clinical manifestations, as follows: category I, GCKD in polycystic kidney disease; category II, hereditary GCKD; category III, syndromic GCKD; category IV, obstructive GCKD; category V, sporadic GCKD [7]. Among the 5 categories, GCKD in polycystic kidney disease and hereditary GCKD were ruled out in this patient due to the absence of cysts in other organs and absence of a family history, respectively. Obstructive GCKD was ruled out due to lack of urinary tract obstruction. Finally, syndromic GCKD was ruled out due to the absence of associated findings, such as hypopigmented macules, angiofibroma, and shagreen patches, which are indicative of tuberous sclerosis [17], and high forehead, epicanthal folds, hypoplasia of supraorbital ridges, and midface, which are indicative of Zellweger syndrome [18]. Therefore, this patient was suspected to have sporadic GCKD. However, there was no history of the primary causes of sporadic GCKD including ischemic damage, such as progressive systemic sclerosis [19], hemolytic uremic syndrome [20], or exposure to drugs [7], such as lithium.

Most subjective and objective symptoms of syndromic or inherited GCKD occur from early childhood. For example, tuberous sclerosis is the most frequent syndromic GCKD, which is found in early childhood. Zellweger syndrome, which is primarily associated with syndromic GCKD, occurs at birth. In addition, inherited GCKD, which is caused by uromodulin disorders, is typically diagnosed in school-age children [21]. However, familiar hypoplastic GCKD, which is caused by mutations in the gene encoding hepatocyte nuclear factor-1β, can occur from fetal stage to the age of 39-years [22]. Results from previous reports indicated that our patient may not express abnormal phenotypes associated with syndromic or inherited diagnosis, even at the age of 30-years. Therefore, we performed genetic testing for *UMOD, MUC1*, and *SEC61A1*, encoding uromodulin, mucin1, and Sec61 translocon alpha 1 subunit. Because no abnormal genetic mutations were detected in this case, we diagnosed the patient with sporadic GCKD.

It is possible to detect any gene mutation in the patient by analyzing the full genome sequence. However, even if we can detect the gene mutations, it is impossible to determine whether the detected gene mutations are responsible for GCKD since the particular gene location has not been identified. The examination of full genome sequences from several families that include individuals with and without GCKD will enable us to determine the mutation responsible for GCKD. Therefore, it is not possible to provide the definitive diagnosis at this time.

Additionally, no mutations of *COL4A3* and *COL4A4* were identified. However, this result is not necessarily incompatible with the diagnosis of TBMN, because mutations of *COL4A3* and *COL4A4* are associated with only 40% of TBMN cases. Thus, many as-yet unknown genes are also responsible for thinning of the GBM [23, 24].

The genetic testing was negative and did not change the outcome or affect treatment in the present case. However, some important information was obtained. First, negative results for commonly associated mutations on the genetic test led to a diagnosis of sporadic GCKD. Second, no known mutations associated with GCKD or TBMN were detected in the present case by genetic testing; therefore, the causal relationship between GCKD and TBMN remains unclear. However, it is possible that both GCKD and TBMN are caused by a single unrecognized mutation. The fact that there was no family history coincides with this hypothesis.

There are other diseases associated with cystic lesions and basement membrane abnormalities, such as hereditary angiopathy with nephropathy, aneurysms, and muscle cramps (HANAC) syndrome. This syndrome is an autosomal dominant syndrome caused by mutations in *COL4A1*, which encodes the α1 chain of collagen IV, a major component of the basement membrane. In this syndrome, renal disease manifests in multicystic kidneys, hematuria in some cases, and decreased eGFR. In addition, renal morphology can indicate basement membrane abnormalities [25, 26]. These features are similar to TBMN accompanied by GCKD, as reported in our case. However, multicystic kidneys associated with renal cysts, not glomerular cysts, and morphological changes in the tubular, vascular, and Bowman’s capsule basement membrane, but not glomerular basement membrane, are found in HANAC syndrome. This is because the α1 chain is a component of tubular, vascular, and Bowman’s capsule basement membranes, but not glomerular basement membranes. Therefore, we consider our case different from HANAC syndrome.

In conclusion, we report the case of TBMN accompanied by sporadic GCKD. TBMN is one of the most frequent causes of isolated microscopic hematuria with no associated physical findings and normal laboratory findings, except hematuria. However, because it is possible that other diseases, such as GCKD, coexist with TBMN, it is important to consider renal biopsy, in addition to conducting serum serological and urine studies. Moreover, because GCKD has 5 categories and multiple etiologies, it is possible that the patient does not yet express the associated abnormal phenotype at the time of diagnosis. Therefore, it is also important to consider genetic testing.

## Data Availability

All data generated during this study are included in this published article.

## Abbreviations

BMI: Body mass index
CT: Computed tomography
eGFR: Estimated glomerular filtration rate
FSGS: Focal segmental glomerulosclerosis
GBM: Glomerular basement membrane
GCKD: Glomerulocystic kidney disease
HANAC: Hereditary angiopathy with nephropathy, aneurysms, and muscle cramps
HPF: High power field
IgA: Immunoglobulin A
RBC: Red blood cell
TBMN: Thin basement membrane nephropathy
